# Physiological characterization of leaf and internode after bud break in Japanese indigenous Koshu grape by comparative RNA sequencing analysis

**DOI:** 10.1371/journal.pone.0194807

**Published:** 2018-03-22

**Authors:** Shinichi Enoki, Yu Hamaguchi, Shunji Suzuki, Hiroyuki Fujisawa, Tomoki Hattori, Kayo Arita, Chiho Yamaguchi, Masachika Mikami, Shu Nagasaka, Keisuke Tanaka

**Affiliations:** 1 Laboratory of Fruit Genetic Engineering, The Institute of Enology and Viticulture, University of Yamanashi, Yamanashi, Japan; 2 NODAI Genome Research Center, Tokyo University of Agriculture, Tokyo, Japan; 3 Department of Agriculture, Tokyo University of Agriculture, Kanagawa, Japan; Universidade de Lisboa Instituto Superior de Agronomia, PORTUGAL

## Abstract

Koshu is indigenous to Japan and considered the most important wine grape in Japan. Koshu grape berry possesses characteristics that make it unique from European *V*. *vinifera* as wine grape. However, the physiological characteristics of Koshu leaf and internode remain unknown. An understanding of those characteristics would contribute to improvements in Koshu cultivation, thereby enhancing grape berry and wine quality. To identify the genes responsible for the physiological characteristics of Koshu, we comprehensively analyzed leaf and internode differences at the transcriptome level between Koshu and Pinot Noir by RNA sequencing. A total of 248 and 131 differentially expressed genes (DEGs) were detected in leaves and internodes, respectively. Gene Ontology (GO) and Kyoto Encyclopedia of Genes and Genomes (KEGG) pathway enrichment analyses of these DEGs revealed that “flavonoid biosynthesis” and “glutathione metabolism” pathways were significantly enriched in Koshu leaves. On the other hand, when internodes were compared, “flavonoid”-related GO terms were specifically detected in Koshu. KEGG pathway enrichment analysis suggested that the expression of such genes as *leucoanthocyanidin reductase* and *flavonol synthase* in the flavonoid biosynthesis pathway was higher in Koshu than Pinot Noir. Measurement of the relative expression levels of these genes by RT-qPCR validated the results obtained by RNA sequencing. The characteristics of Koshu leaf and internode, which are expected to produce flavonoids with antibacterial activity and UV protection function, would suit Japanese climate as a survival strategy.

## Introduction

Koshu is an indigenous Japanese *Vitis* species mainly cultivated in Yamanashi Prefecture in Japan. Koshu was recognized as a wine grape cultivar in 2010 by the International Organization for Vine and Wine (OIV) and its passport data are registered in *Vitis* International Variety Catalogue (VIVC) [1, 2]. Although Koshu is still considered the official grape cultivar for winemaking, its genetic background is suggested to consist of 70% European *V*. *vinifera* and 30% East Asian wild *Vitis* species [3]. Koshu grape is the most important hybrid variety for white wine making in Japan [4–7].

Koshu berry has certain characteristics that distinguish it from European *V*. *vinifera* berry. Koshu berry size is more than twice those of *V*. *vinifera* cvs. Chardonnay, Sauvignon Blanc, Merlot, Cabernet Sauvignon, and Pinot Noir, which are famous European wine grape cultivars. Koshu berry skin shows a light pink color because the skin moderately accumulates anthocyanin pigments unlike major European white wine grape cultivars [8]. Morphological research of Koshu bunch and berry [9] and chemical analysis of berry metabolites related to wine quality [10–12] had been conducted.

The physiological characteristics of Koshu grapevine, particularly the leaf and the internode, have remained unknown unlike berry, because there are no studies comparing Koshu and major European wine grape cultivars except one that compares anthocyanin composition in leaf at harvest among Koshu and various cultivars [13]. The quality of grape berry as the raw material of wine depends largely on physiological characteristics, such as shoot (leaf and internode) growth of grapevine [14, 15] and adaptability to meteorological conditions [12, 16]. Therefore, clarifying the physiological characteristics of Koshu leaf and internode is important for the development of new cultivation techniques and the accumulation of basic breeding information suitable for its characteristics.

Recent research using transcriptome analysis, such as RNA sequencing (RNA-seq), is rapidly gaining momentum in an effort to reveal the molecular mechanisms underlying the physiological characteristics of fruit [17]. In grapevine, transcriptome analysis was conducted to reveal growth mechanisms at different developmental stages [18–22] or in response to environmental stress [23, 24] and to detect genes expressed by different cultivars [25–27]. Unlike microarray technology, RNA-seq is advantageous in that it is possible to analyze a transcriptome over a wide dynamic range from low to high expression levels and to discover unknown genes. Therefore, in this study, we used RNA-seq as one of the outstanding methods to physiologically characterize Koshu.

To clarify the physiological characteristics of Koshu leaf and internode at the transcriptome level, we performed comprehensive and comparative gene expression analysis of Japanese Koshu and European Pinot Noir using RNA-seq. We found that Koshu leaf and internode exhibit specific characteristics, namely, flavonoid biosynthesis and glutathione metabolism, and discussed the significance of these characteristics in viticulture and winemaking.

## Materials and methods

### Plant materials

Grapevines of Koshu and Pinot Noir were grown in the experimental vineyard of The Institute of Enology and Viticulture, University of Yamanashi, Yamanashi Prefecture, Japan. Three shoots per plant species were sampled on day 7 after bud break. The shoots were separated into leaves and internodes. Each sample was described as follows: Pinot Noir leaves (PL), Pinot Noir internodes (PI), Koshu leaves (KL), and Koshu internodes (KI). These samples were frozen in liquid nitrogen and stored at -80 °C until use.

### Total RNA extraction and qualification

The leaves and internodes of each plant species were homogenized in an SK mill (SK-200, Tokken, Kashiwa, Japan) after freezing with liquid nitrogen. Total RNA extraction from the pulverized samples was performed with a NucleoSpin RNA Plant Kit (TaKaRa, Otsu, Japan) after pretreatment with a Fruit-mate for RNA Purification (TaKaRa) according to the manufacturer’s instructions.

### Construction of cDNA library

Total RNA quality and quantity were evaluated with an Agilent 2100 Bioanalyzer (Agilent Technologies, Santa Clara, CA, USA) and an Agilent RNA 6000 Nano Kit (Agilent Technologies). Library preparation was performed by using a NEBNext Ultra RNA Library Prep Kit for Illumina (New England Biolabs, Ipswich, MA, USA), according to the protocol. Briefly, oligo-(dT) magnetic beads were used to isolate poly-(A) mRNA from total RNA and a fragmentation buffer was added to cut mRNA into short fragments. Using these short fragments as templates, first-strand cDNAs were synthesized using random hexamer primers and SuperScript III Reverse Transcriptase (Invitrogen, Carlsbad, CA, USA). After second-strand cDNA synthesis, end-repaired and dA-tailed fragments were connected to sequencing adapters. The adapter-ligated cDNA fragments were amplified by 15 cycles of PCR and the products were cleaned up by using AMPure XP magnetic beads (Beckman Coulter, Brea, CA, USA). Library quality and concentration were assessed using the Agilent 2100 Bioanalyzer and an Agilent DNA 1000 Kit (Agilent Technologies). More precise concentration of the libraries was determined using a Step One Plus Real-Time PCR System (Applied Biosystems Laboratories, Foster City, CA, USA) and a KAPA Library Quantification Kit (Kapa Biosystems, Wilmington, MA, USA).

### Transcriptome sequencing

All libraries were diluted to 10 nM concentration and then mixed in an equal amount. The library mixture was sequenced by 1 × 100 bp single read sequencing using an Illumina HiSeq 2500 (Illumina, San Diego, CA, USA). Reads in FASTQ format were generated using bcl2fastq2 Conversion Software (Illumina, version 2.18). The read data were submitted to the DDBJ Read Archive (Accession number DRA005833).

### Differentially expressed gene analysis

Adapter sequences and the initial 13 bases in each read were removed using CLC Genomics Workbench 9.0 (Qiagen, Hilden, Germany). Then, the premeasured read data were mapped to the reference genome of *V*. *vinifera* genotype (PN40024) that was derived from Pinot Noir in wine grape cultivar that retrieved from the NCBI genome database (https://www.ncbi.nlm.nih.gov/). The genome of PN40024 had been sequenced [28]. Mapping parameters were as follows: (i) mismatch cost, 2; (ii) insertion cost, 3; (iii) deletion cost, 3; (iv) length fraction, 0.8; (v) and similarity fraction, 0.8. The expression levels for each sample were calculated from mapping-based count data and expressed as “Total counts”. Differentially expressed genes (DEGs) used to compare the expression levels between Koshu and Pinot Noir in each tissue were detected using False Discovery Rate (FDR) (q < 0.05) after edgeR.

### Enrichment analysis

Gene Ontology (GO) and Kyoto Encyclopedia of Genes and Genomes (KEGG) pathway enrichment analyses of gene sets were performed using the web-based tool DAVID 6.8 (https://david.ncifcrf.gov). The enriched GO terms and pathways were statistically analyzed using the modified Fisher’s exact test (p < 0.05) contained in the tool.

### Pathway analysis

KEGG metabolic maps for the enriched pathways were highlighted using fold change from normalized expression levels by transcripts per million (TPM) with the R package “pathview” [29] to compare expression levels in leaf and internode between Koshu and Pinot Noir.

### Real-time RT-PCR validation

First-strand cDNA was synthesized from total RNA with a PrimeScript RT Reagent Kit with gDNA Eraser (Perfect Real Time) (TaKaRa). Real-time RT-PCR (RT-qPCR) was performed with an SYBR Premix Ex Taq II (TaKaRa) by using the standard curve method on Thermal Cycler Dice Real Time System Single Software ver. 5.10A (TaKaRa). The RT-qPCR conditions were as follows: 37 °C for 15 min for RT reaction and 85 °C for 5 s for cDNA synthesis, and then 40 cycles at 95 °C for 5 s and at 60 °C for 30 s for PCR amplification. Nucleotide sequences of the primers used in this study are listed in S1 Table. Actin was used for normalization because it had been recommended as a reference gene in grapevine [30]. The expression level of each gene was expressed as a relative value to actin. RT-qPCR validation data were expressed as means ± standard error. Statistical analysis was performed by the Student’s *t*-test with Excel statistics software 2012 (Social Survey Research Information, Tokyo, Japan).

## Results

### Summary of cDNA sequencing and mapping

S2 and S3 Tables show summaries of sequencing and mapping, respectively. A total of 12 cDNA libraries were constructed from the total RNA of each sample (KL1-3, KI1-3, PL1-3, and PI1-3). Approximately 23–30 million raw reads were generated from the libraries by sequencing with an Illumina HiSeq 2500 system (S2 Table). All data showed Q30 or higher base quality of 94% and mean quality score of Q37 through the pass filter. After filtering, approximately 23–30 million clean reads remained (S3 Table). These reads were mapped to Pinot Noir reference genome. The mapping efficiencies were in the range of 83.07–89.11%.

### Differentially expressed gene analysis

**Fig 1:**
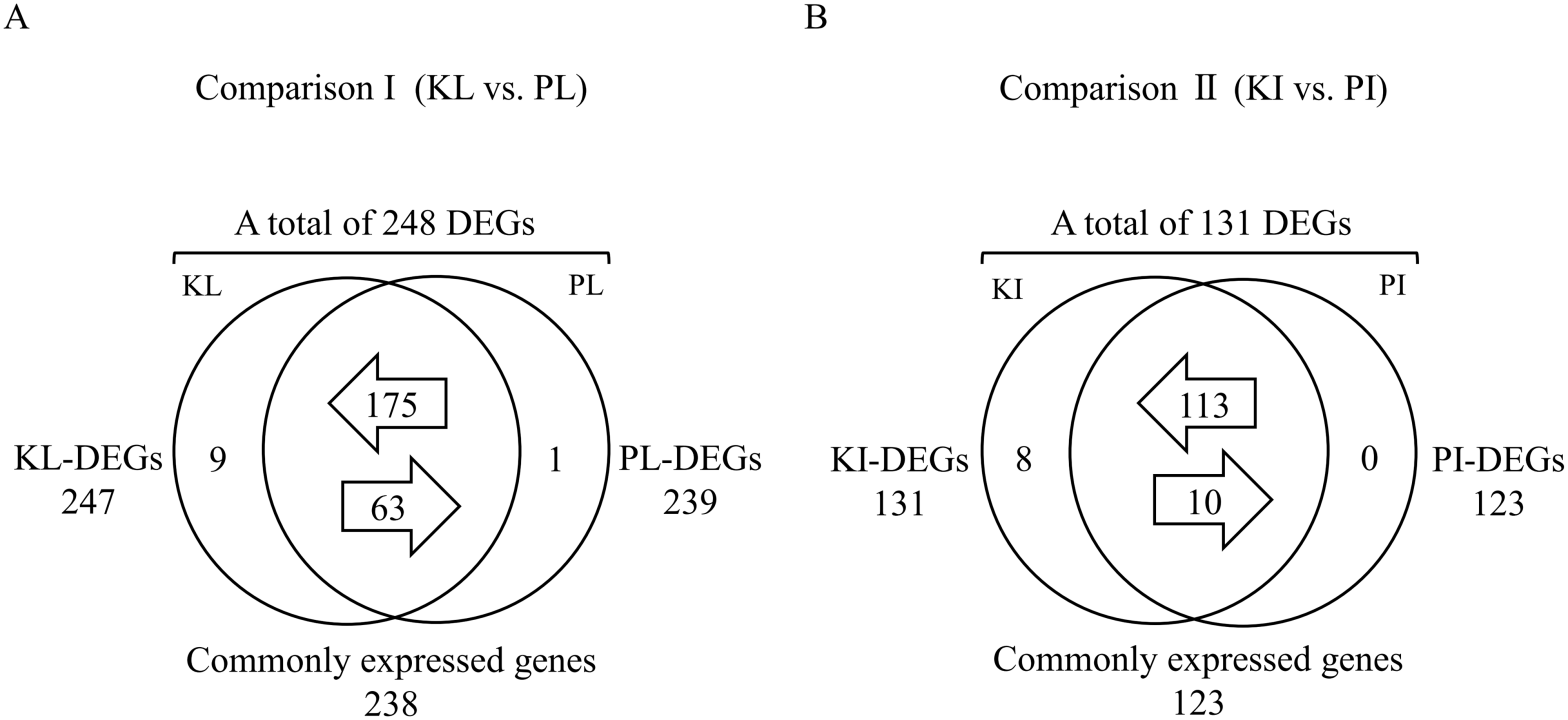
Number of differentially expressed genes in Comparison I (KL vs. PL) (A) and Comparison II (KI vs. PI) (B). The total number of DEGs for each sample and the number of DEGs for each sample (KL-DEGs, PL-DEGs, KI-DEGs, and PI-DEGs) are shown outside Venn diagrams. Of these, the number of genes that are specifically expressed only in either sample (defined as -specific expressed genes) and the number of genes that are commonly expressed in both samples (commonly expressed genes) are shown. Of these commonly expressed genes, the number of genes with higher expression levels in either sample (-highly expressed genes) is shown in the arrows. DEGs were detected by using FDR (q < 0.05) after edgeR. KL: Koshu leaves, PL: Pinot Noir leaves, KI: Koshu internodes, PI: Pinot Noir internodes.

Venn diagrams in Fig 1A and 1B show the number of DEGs in Koshu and Pinot Noir leaves (Comparison I) and internodes (Comparison II), respectively. In Comparison I (KL vs. PL) of Fig 1A and S4 Table, there were a total of 248 DEGs in KL and PL. Of these, 247 DEGs existed in KL (defined as KL-DEGs) whereas 239 DEGs existed in PL (PL-DEGs). Nine genes were expressed specifically only in KL (defined as KL-specific expressed genes), 238 genes were commonly expressed in KL and PL, and one gene was expressed specifically only in PL (PL-specific expressed gene). Of the 238 genes that were commonly expressed in KL and PL, 175 genes were more highly expressed in KL than PL (defined as KL-highly expressed genes) and 63 genes were more highly expressed in PL than KL (PL-highly expressed genes). In Comparison II (KI vs. PI) of Fig 1B and S5 Table, there were a total of 131 DEGs in KI and PI. Of these, 131 DEGs existed in KI (defined as KI-DEGs) whereas 123 DEGs existed in PI (PI-DEGs). There were eight KI-specific expressed genes and 123 commonly expressed genes in KI and PI. No PI-specific expressed gene was detected. Of the 123 commonly expressed genes, 113 were KI-highly expressed genes and 10 were PI-highly expressed genes. Many of the detected Koshu-specific expressed and Koshu-highly expressed genes had unknown functions. The number of DEGs in Koshu and Pinot Noir was relatively small, suggesting that the basic physiological functions of these two species are almost the same and the physiological characteristics of Koshu are regulated by a relatively small number of DEGs.

### Enrichment analysis

Tables 1 and 2 show the results of enrichment analyses of Koshu and Pinot Noir DEGs. GO enrichment analysis was conducted to identify the major biological functions of the DEGs (Table 1). None of the DEGs classified into the cellular component category were detected in any of the comparisons. In Comparison I, the number of significantly enriched GO terms was six in the biological process (BP) category and 12 in the molecular function (MF) category in KL-DEGs (KL-specific expressed and KL-highly expressed genes). It was also six in the BP category and two in the MF category in PL-DEGs (PL-specific expressed and PL-highly expressed genes). In Comparison II, there were eight GO terms in the BP category and 10 in the MF category in KI-DEGs (KI-specific expressed and KI-highly expressed genes). None of the DEGs classified into any GO term were detected in PI-DEGs (PI-highly expressed genes). The significantly enriched GO terms in both KL and KI included “secondary metabolic process” and “secondary metabolite biosynthetic process” in the BP category, and “oxidoreductase activity, acting on paired donors, with incorporation or reduction of molecular oxygen” in the MF category. As specific terms related to secondary metabolism, terms related to glutathione (“glutathione metabolic process” in the BP category and “glutathione transferase activity” in the MF category) in KL and to flavonoid (“flavonoid metabolic process”, “flavonoid glucuronidation”, and “flavonoid biosynthetic process” in the BP category and “quercetin 3-O-glucosyltransferase activity” and “quercetin 7-O-glucosyltransferase activity” in the MF category) in KI were significantly detected.

**Table 1:** GO enrichment analysis.

Comparison		Category	Term	P-Value
**I**	**KL-DEGs**	**BP**	cellular amide metabolic process	1.40E-02
			peptide metabolic process	1.20E-02
			secondary metabolic process	2.40E-02
			cellular modified amino acid metabolic process	7.80E-03
			glutathione metabolic process	4.30E-03
			secondary metabolite biosynthetic process	3.60E-02
		**MF**	ion binding	7.90E-03
			cation binding	8.90E-03
			metal ion binding	8.80E-03
			transition metal ion binding	1.80E-02
			iron ion binding	2.10E-05
			oxidoreductase activity, acting on paired donors, with incorporation or reduction of molecular oxygen	3.00E-05
			tetrapyrrole binding	5.30E-04
			heme binding	1.80E-03
			monooxygenase activity	5.50E-04
			glutathione transferase activity	5.30E-03
			oxidoreductase activity, acting on paired donors, with incorporation or reduction of molecular oxygen, NAD(P)H as one donor, and incorporation of one atom of oxygen	2.70E-02
			transferase activity, transferring alkyl or aryl (other than methyl) groups	2.30E-02
**I**	**PL-DEGs**	**BP**	aromatic compound biosynthetic process	4.00E-02
			cellular amino acid catabolic process	4.80E-02
			cinnamic acid biosynthetic process	2.10E-02
			cinnamic acid metabolic process	2.10E-02
			L-phenylalanine catabolic process	2.30E-02
			L-phenylalanine metabolic process	3.40E-02
		**MF**	ammonia-lyase activity	4.20E-02
			phenylalanine ammonia-lyase activity	3.50E-02
**II**	**KI-DEGs**	**BP**	secondary metabolic process	1.40E-02
			uronic acid metabolic process	2.70E-02
			secondary metabolite biosynthetic process	4.50E-02
			glucuronate metabolic process	2.70E-02
			flavonoid metabolic process	3.20E-02
			flavonoid glucuronidation	2.70E-02
			flavonoid biosynthetic process	3.10E-02
			cellular glucuronidation	2.70E-02
		**MF**	enzyme inhibitor activity	2.50E-03
			enzyme regulator activity	2.30E-02
			molecular function regulator	2.90E-02
			oxidoreductase activity, acting on paired donors, with incorporation or reduction of molecular oxygen	3.40E-02
			quercetin 3-O-glucosyltransferase activity	4.80E-02
			quercetin 7-O-glucosyltransferase activity	4.80E-02
			endopeptidase inhibitor activity	4.80E-02
			endopeptidase regulator activity	4.80E-02
			peptidase inhibitor activity	4.80E-02
			peptidase regulator activity	4.80E-02

KL: Koshu leaves, PL: Pinot Noir leaves, KI: Koshu internodes, BP: biological process, MF: molecular function.

**Table 2:** KEGG pathway enrichment analysis.

Comparison		Term	P-Value
**I**	**KL-DEGs**	Flavonoid biosynthesis	1.80E-03
		Glutathione metabolism	4.50E-02
	**PL-DEGs**	Biosynthesis of secondary metabolites	1.00E-02

KL: Koshu leaves, PL: Pinot Noir leaves.

KEGG pathway enrichment analysis was conducted to identify the biological metabolic pathway that includes these DEGs (Table 2). In Comparison I (KL vs. PL), two pathways, including “flavonoid biosynthesis” and “glutathione metabolism”, in KL, and one pathway, “biosynthesis of secondary metabolites”, in PL were significantly enriched. In Comparison II (KI vs. PI), none of the DEGs classified into any of the pathways were detected. These results by GO and KEGG pathway enrichment analyses indicated that flavonoid and glutathione pathways physiologically characterize Koshu leaf and internode.

### Pathway analysis

**Fig 2:**
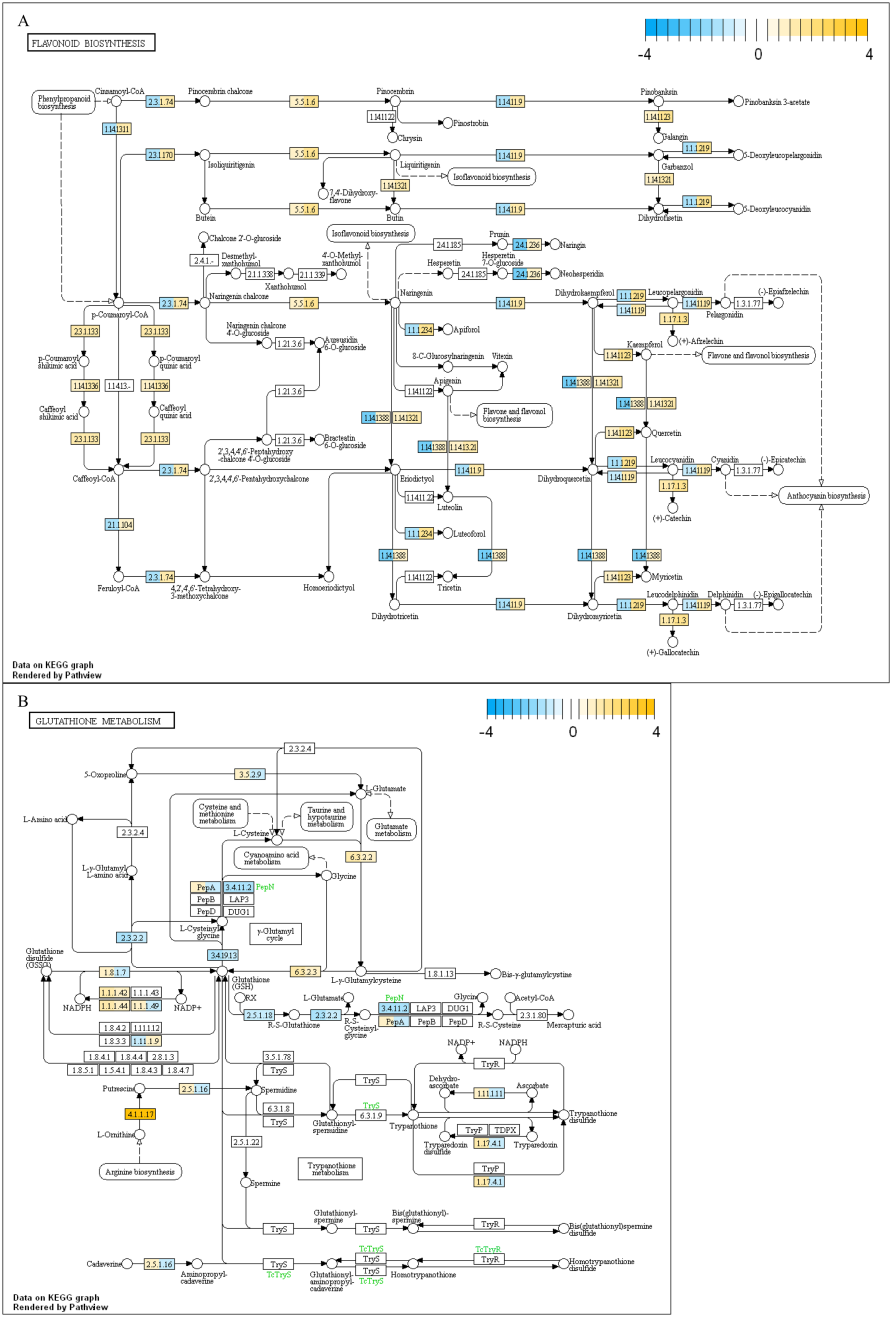
Transcriptional profiles in flavonoid biosynthesis and glutathione metabolism pathway maps derived from KEGG database. (A) Flavonoid biosynthesis pathway. (B) Glutathione metabolism pathway. Color in the rectangular box indicates relative gene expression levels (left half, leaf; right half, internode). The numerical value bar at the upper right corner shows the coloring method using the log2 fold change (Koshu/Pinot Noir) of expression levels by transcripts per million (TPM) between Koshu and Pinot Noir.

To compare gene expression levels between Koshu and Pinot Noir in the significantly enriched metabolic pathways in Table 2, all gene expression levels contained in these pathways were mapped to the KEGG metabolic pathways (Fig 2). Fig 2A and 2B show flavonoid biosynthesis (KEGG PATHWAY: vvi00941) and glutathione metabolism (KEGG PATHWAY: vvi00480) pathways, respectively. Numbers in boxes in Fig 2 correspond to Enzyme Commission (EC) numbers. A single gene or gene family encoding each reaction system is denoted as gene product [EC number]. In the flavonoid biosynthesis pathway (Fig 2A), the six genes encoding leucoanthocyanidin reductase (LAR) [EC: 1.17.1.3], flavonoid 3'-monooxygenase (F3’H) [EC: 1.14.13.21], chalcone isomerase (CHI) [EC: 5.5.1.6], shikimate O-hydroxycinnamoyltransferase [EC: 2.3.1.133], coumaroylquinate (coumaroylshikimate) 3'-monooxygenase [EC: 1.14.13.36], and flavonol synthase (FLS) [EC: 1.14.11.23] tended to be more highly expressed in Koshu than Pinot Noir. In the glutathione metabolism pathway (Fig 2B), the five genes encoding isocitrate dehydrogenase [EC: 1.1.1.42], 6-phosphogluconate dehydrogenase [EC: 1.1.1.44, 1.1.1.343], ornithine decarboxylase (ODC) [EC: 4.1.1.17], glutamate-cysteine ligase [EC: 6.3.2.2], and glutathione synthase [EC: 6.3.2.3] also tended to be more highly expressed in Koshu than Pinot Noir. In the flavonoid biosynthesis pathway, unlike the glutathione metabolism pathway, all gene expression levels found in grapevine were observed in both Koshu and Pinot Noir.

### RT-qPCR validation

**Fig 3:**
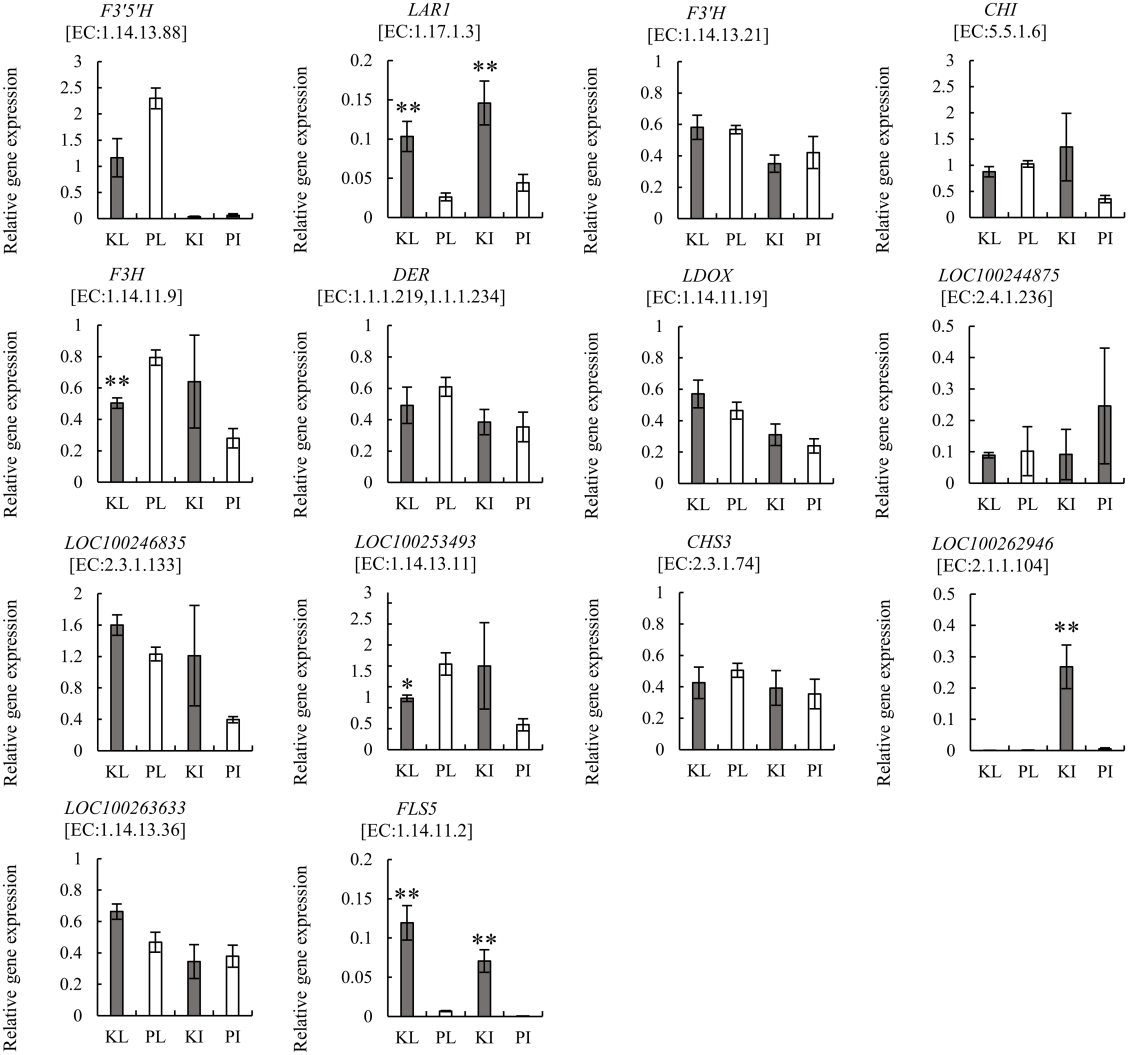
RT-qPCR validation to confirm results of RNA-seq analysis. The expression of 14 selected genes in the flavonoid biosynthesis pathway relative to that of actin gene was calculated. Each gene encoding an enzyme is denoted as [EC number]. Bars indicate standard error for three biological replicates and two technical replicates. * and ** indicate significant difference compared with control (Pinot Noir) at *P* < 0.05 and *P* < 0.01, respectively. KL: Koshu leaves, PI: Pinot Noir leaves, KI: Koshu internodes, PI: Pinot Noir internodes.

To confirm the results of RNA-seq, RT-qPCR validation was conducted (Fig 3). We focused on the flavonoid biosynthesis pathway where many DEGs tend to exist in Koshu and Pinot Noir (Fig 2A). Fourteen genes were selected for this validation. The relative expression levels of most of the selected genes showed a similar trend to Fig 2 except for those encoding naringenin 3-dioxygenase (F3H) [EC: 1.14.11.9] and trans-cinnamate 4-monooxygenase [EC: 1.14.13.11] in KL. The expression of genes encoding LAR [EC: 1.17.1.3], caffeoyl-CoA O-methyltransferase (CCOAMT) [EC: 2.1.1.104] (only in KI), and FLS [EC: 1.14.11.23] in Koshu was consistently and significantly higher than that in Pinot Noir (Tables 1 and 2 and Figs 2 and 3). These results indicated the validity of the results obtained by RNA-seq.

## Discussion

**Fig 4:**
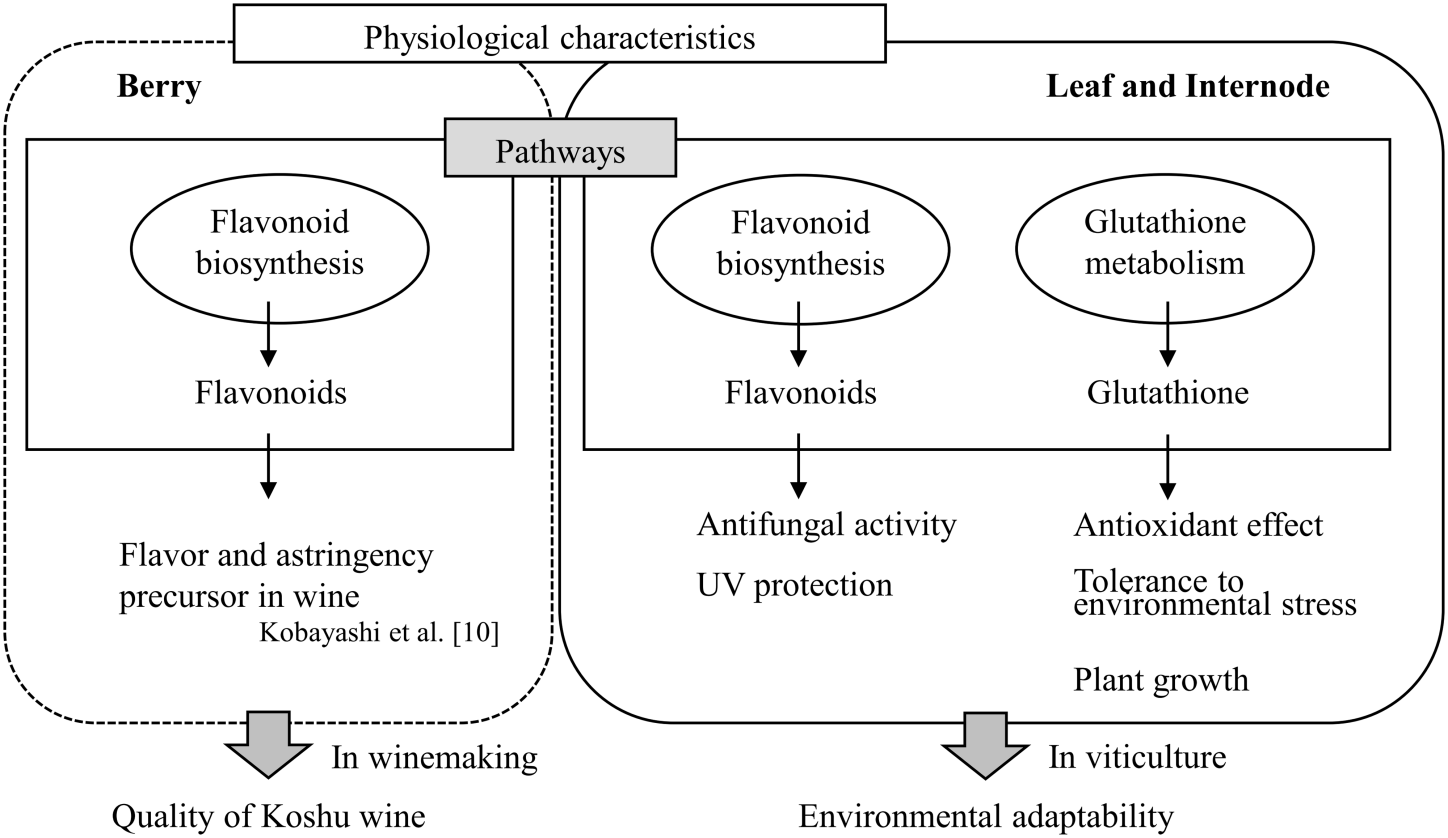
Proposed model of physiological characteristics of Koshu leaf and internode.

A model of the physiological characteristics of Koshu proposed in this study is shown in Fig 4. Here, we revealed that flavonoid biosynthesis and glutathione metabolism pathways were the unique characteristics of Koshu leaf and internode. The high expression of *FLS*, *LAR*, and *CCOAMT* in the flavonoid biosynthesis pathway in Koshu leaf and internode (Fig 2A) indicated that Koshu has high flavonoid content. Flavonoids are known to be widely distributed in leaf and internode and to exhibit antifungal activity [31, 32] and ultraviolet light (UV)-protective activity [33, 34] in plants. Yamanashi Prefecture, the main cultivation area of Koshu in Japan, has high temperature and humidity. For example, Yamanashi Prefecture usually receives approximately 850 mm of rainfall in the growing season. The accumulated monthly rainfall during the growing season (from April to October) in Yamanashi Prefecture changes markedly due to the start of the rainy season in early summer and irregular typhoons [11]. Under such meteorological conditions, phytopathogens generally proliferate easily, causing various diseases in grapevine. UV radiation damages DNA, alters the photosynthetic machinery, and adversely affects plant growth and morphogenesis [35]. In the case of *V*. *vinifera* cv. Tempranillo which is indigenous to Spain, its high UV tolerance seems to be due to its geographical origin in Mediterranean climate that is characterized by relatively high solar radiation [36]. The physiological characteristics of Koshu leaf and internode might offer a superior advantage as a survival strategy in regions less suitable for viticulture.

In winemaking, some flavonoids are known to give an astringent taste and a unique flavor to wine. Kobayashi et al. [10] reported that the contents of flavonoids, including monomeric flavonoids (quercetin derivatives) and hydroxycinnamic acids (p-caftaric and p-coutaric acids), were higher in Koshu berry than European red and white wine grape cultivars. FLS is involved in the synthesis of quercetin. The genes encoding shikimate O-hydroxycinnamoyltransferase, coumaroylquinate (coumaroylshikimate) 3'-monooxygenase, and CCOAMT are involved in the synthesis of hydroxycinnamic acids (coumaric and caffeic acids as precursors of p-caftaric and p-coutaric acids, respectively). Taken together, the results indicate that whole organs of Koshu grapevine, including berry, leaf, and internode, may accumulate high levels of flavonoids compared with other *V*. *vinifera* cultivars. This characteristic of Koshu upon genetic variation may be due to the fact that Koshu is not a pure *Vitis vinifera* cultivar but a hybrid variety. The high total phenolic content and the slight astringency of Koshu wines are attributed to the transcriptional characteristics of Koshu grapevine. To improve the quality of Koshu wines, various enological techniques, such as hyperoxidation [5] and skin contact [6], have been adopted. The hyperoxidation method is used to remove excess red blush and astringency produced by phenolic compounds derived from grape skin, which are often regarded as a defect of white wine. On the other hand, the skin contact method involves holding the skin and the juice together before the fermentation process and extracting such components as phenolic compounds from the skin. This technique increases richness, complexity, and aromatic intensity of white wine by using the components derived from grape skin.

The antioxidant glutathione is involved in the detoxification of reactive oxygen species, thereby protecting plant from oxidative damage. The antioxidant function of glutathione confers tolerance to environmental stress, such as salt, high/low temperature, and toxic metal, in plant [37]. The high glutathione content in Koshu leaf and internode, which is predicted from the high expression of *glutamate—cysteine ligase* and *glutathione synthase* in Fig 2B, suggests that Koshu leaf and internode adapt to environmental stress under vineyard conditions. The application of exogenous glutathione to plants promoted growth and increased harvest index [38]. As Koshu leaf and internode show great vigor, the traditional shelf style (overhead trellis), but not Guyot style, is used for Koshu cultivation. The vigorous growth of Koshu leaf and internode might be due to physiological characteristics related to the high glutathione contents in leaf and internode.

This study provided comprehensive and comparative transcriptomic information of Koshu leaf and internode after bud break using RNA-seq. The number of DEGs in Koshu and Pinot Noir is relatively small (Fig 1, S4 and S5 Tables), suggesting that the basic physiological characteristics of the two species might be identical. RNA-seq detected differences in the transcriptional levels of a small number of DEGs between the two species (Tables 1 and 2, and Fig 2), which are related to important physiological traits in viticulture and winemaking (Fig 4). Therefore, comprehensive and comparative transcriptomic analysis using RNA-seq is expected to clearly capture differences in transcriptional levels between grapevine species. Comparative analysis of grapevine cultivars at the transcriptome level [24–26] has provided fundamental information for improving berry quality and made it possible to use the target cultivar as a genetic resource. In this study, we demonstrated that flavonoid biosynthesis and glutathione metabolism pathways are the physiological characteristics of Koshu leaf and internode. To breed new hybrid varieties that can adapt to Japanese meteorological conditions, the development of molecular markers based on the physiological characteristics of Koshu leaf and internode is required.

## Supporting information

S1 Table Nucleotide sequences of primers used for RT-qPCR. (XLSX)

S2 Table Summary of cDNA sequencing. KL: Koshu leaves, KI: Koshu internodes, PL: Pinot Noir leaves, PI: Pinot Noir internodes.
(XLSX)

S3 Table Efficiency of mapping to grape reference genome. KL: Koshu leaves, KI: Koshu internodes, PL: Pinot Noir leaves, PI: Pinot Noir internodes.
(XLSX)

S4 Table DEG list in Comparison I (KL vs. PL). Ch: chromosome, KL: Koshu leaves, PL: Pinot Noir leaves, FDR: False discovery rate, TPM: Transcripts per million.
(XLSX)

S5 Table DEG list in Comparison II (KI vs. PI). Ch: chromosome, KI: Koshu internodes, PI: Pinot Noir internodes, FDR: False discovery rate, TPM: Transcripts per million.
(XLSX)
